# Cross‐Cultural Adaptation and Psychometric Evaluation of the Persian Cognitive Assessment Scale for Stroke Patients: A Cross‐Sectional Study

**DOI:** 10.1002/hsr2.73260

**Published:** 2026-09-15

**Authors:** Farzad Hekmatifar, Nazila Akbarfahimi, Mohammad Sayadnasiri, Razieh Bidhendi‐Yarandi, Samira Rostami, Negin Fallahi‐Khoshknab, Charles Benaim

**Affiliations:** ^1^ Department of Occupational Therapy University of Social Welfare and Rehabilitation Sciences Tehran Iran; ^2^ Department of Occupational Therapy, School of Rehabilitation Sciences, Clinical Research Development Center of Rofeideh Rehabilitation Hospital University of Social Welfare and Rehabilitation Sciences Tehran Iran; ^3^ School of Behavioral Sciences and Mental Health, Rofeideh Rehabilitation Hospital, Razi Educational and Therapeutric Center University of Social Welfare and Rehabilitation Sciences Tehran Iran; ^4^ Social Determinants of Health Research Center University of Social Welfare and Rehabilitation Sciences Tehran Iran; ^5^ Department of Biostatistics and Epidemiology, School of Social Health University of Social Welfare and Rehabilitation Sciences Tehran Iran; ^6^ Rofeideh Rehabilitation Hospital University of Social Welfare and Rehabilitation Sciences Tehran Iran; ^7^ University Hospital of Lausanne (CHUV) Lausanne Switzerland; ^8^ CRR-Suva Sion Switzerland

**Keywords:** aphasia, cognitive assessment scale for stroke patients, cognitive dysfunction, reliability, stroke, validity

## Abstract

**Background and Aims:**

This study aimed to translate, culturally adapt, and evaluate the psychometric properties of the Persian Cognitive Assessment Scale for Stroke Patients (P‐CASP).

**Methods:**

In this cross‐sectional psychometric study, 136 individuals with stroke and 60 age‐ and sex‐matched healthy controls were enrolled. Translation and cultural adaptation followed the Beaton and Dewey protocol. Content validity was assessed using CVR and CVI based on ratings from 20 experts. Concurrent validity was assessed using Pearson's correlation with the MMSE, and known‐groups validity was examined by comparing stroke and control groups. Test–retest reliability was assessed in 76 participants over 2 weeks. Internal consistency was estimated using Guttman's lambda‐2 (*λ*2). Measurement error was assessed using MDC_95_, and ROC analysis evaluated discrimination between groups.

**Results:**

CVR and CVI ranged from 0.70 to 1.00 and 0.95–1.00, respectively. P‐CASP scores correlated strongly with MMSE scores (*r* = 0.886, *p *< 0.001). Known‐groups validity was supported by a significant between‐group difference (*t* = 7.508, *p *< 0.001; Cohen's *d* = 1.36). Internal consistency was high (*λ*2 = 0.922), and test–retest reliability was excellent (ICC = 0.997, 95% CI: 0.996–0.998). MDC_95_ was 1.66 points. ROC analysis showed excellent discrimination (AUC = 0.980, 95% CI: 0.93–0.98); a cutoff of 31.25 yielded 100% sensitivity and 73.7% specificity.

**Conclusion:**

The P‐CASP demonstrated strong validity, high internal consistency, excellent test–retest reliability, and good discriminative performance. It may provide a practical cognitive assessment tool for individuals with stroke, including those with aphasia. Further factor‐analytic and longitudinal studies are warranted.

## 1 Introduction

Cognitive impairment is a common and clinically important consequence of stroke and may affect survivors during the early stages of recovery. Post‐stroke cognitive deficits can involve multiple domains, including attention, memory, executive function, language, visuospatial abilities, and praxis, and may interfere with participation in rehabilitation and everyday activities [1, 2, 3, 4]. Early identification of cognitive difficulties is therefore an important component of comprehensive stroke care and may help clinicians identify individuals who require more detailed cognitive evaluation and appropriate rehabilitation planning [2, 4].

The Mini‐Mental State Examination (MMSE) and Montreal Cognitive Assessment (MoCA) are widely used for cognitive screening. However, their interpretation in people with stroke may be complicated by language and communication impairments, particularly aphasia, as well as by visuospatial and other neurological deficits that may interfere with task performance [5, 6]. Although the MoCA is generally more sensitive than the MMSE for detecting mild cognitive impairment and executive dysfunction, both instruments require varying degrees of verbal comprehension and/or verbal response. Consequently, performance may partly reflect language‐related limitations rather than cognitive ability in individuals with post‐stroke aphasia [5, 6].

Stroke‐specific cognitive screening instruments have subsequently been developed to address some of these limitations. The Oxford Cognitive Screen (OCS), for example, was specifically developed for people with stroke and incorporates tasks designed to reduce the influence of aphasia and visuospatial neglect [7]. Another stroke‐specific instrument, the Cognitive Assessment Scale for Stroke Patients (CASP), was developed as a brief bedside measure of cognitive functioning with reduced reliance on verbal responses [5, 8, 9]. The CASP assesses several cognitive domains relevant to stroke, including praxis, short‐term memory, temporal orientation, executive function, visuospatial neglect, and language. Previous studies of the original and translated versions of the CASP have reported promising evidence regarding its validity and reliability, including its applicability in individuals with language impairment [5, 9, 10]. However, the availability of a psychometrically evaluated instrument in a specific language and cultural context cannot be assumed solely on the basis of evidence from other language versions.

Collectively, these features support the CASP's practicality and favorable psychometric performance, particularly during the acute and subacute phases following stroke [5, 9]. Although Persian versions of general cognitive screening tools, such as the MMSE and MoCA, are available, no validated Persian version of the CASP—a stroke‐specific cognitive screening instrument specifically designed to reduce the influence of aphasia and language impairment—has been available. Given the widespread use of Persian across Iran, Afghanistan, and Tajikistan, and the need for a culturally appropriate stroke‐specific cognitive assessment tool for individuals with post‐stroke language deficits, cross‐cultural adaptation and psychometric evaluation of a Persian version were warranted. Therefore, the present study aimed to translate, culturally adapt, and evaluate the validity and reliability of the Persian version of the CASP (P‐CASP).

## 2 Methods

### 2.1 Study Methodology and Location

This methodological study was conducted in two phases: (1) translation and cross‐cultural adaptation of the CASP into Persian, and (2) evaluation of the preliminary psychometric properties of the Persian version (P‐CASP). The psychometric evaluation included assessment of content validity, concurrent validity, known‐groups validity, internal consistency, test–retest reliability, and measurement error. Receiver operating characteristic (ROC) curve analysis was additionally performed to examine the ability of the P‐CASP to discriminate between the stroke and healthy control groups included in the study.

Participants were recruited between 2024 and 2025 using non‐probability convenience sampling from three neurology‐affiliated rehabilitation hospitals and centers in Tehran, Iran.

### 2.2 Participants

A total of 136 people with stroke (PwS) were enrolled in the psychometric evaluation phase. Eligible participants had experienced a first‐ever unilateral ischemic stroke within the previous 3 months and were between 40 and 60 years of age. This age range was selected to reduce the potential influence of age‐related cognitive decline during the initial psychometric evaluation.

Participants were excluded if they had a history of psychiatric disorders, psychosis, other neurological diseases that could affect cognitive function, significant pre‐stroke visual impairment, or documented pre‐stroke cognitive impairment. Hemorrhagic stroke was excluded to maintain a clinically homogeneous sample and reduce variability associated with different stroke pathophysiology and recovery patterns.

Participants were initially screened according to the eligibility criteria. Depressive symptoms were subsequently assessed using the 13‐item short form of the Beck Depression Inventory (BDI‐II). A predefined cutoff score of < 8 was applied to minimize the potential influence of clinically significant depressive symptoms on cognitive test performance [11].

Demographic and clinical characteristics, including age, sex, educational level, time since stroke onset, stroke location, and aphasia status, were recorded using participant interviews and medical records. Aphasia status was determined based on the diagnosis documented by the treating physician.

#### 2.2.1 Healthy Control Group

For discriminant validity analysis, a subset of 60 stroke participants was selected from the total sample of 136 using age‐ and gender‐matched pair sampling, corresponding to the 60 healthy controls. This approach was adopted to ensure strict demographic comparability between groups and to minimize the potential confounding effects of age and gender on between‐group score differences. These participants had no history of cognitive disorders or psychiatric medication use. Moreover, educational level was also recorded to examine potential group differences as a confounding variable.

### 2.3 Ethical Approval

The study was conducted in accordance with the principles of the Declaration of Helsinki. Ethical approval was obtained from the Ethics Committee of the University of Social Welfare and Rehabilitation Sciences (Approval Code: IR.USWR.REC.1402.260).

All participants were informed about the objectives and procedures of the study before enrollment. Written informed consent was obtained from all participants or, when applicable, from their legally authorized representatives.

As this was a methodological psychometric study involving translation, cultural adaptation, and evaluation of measurement properties, prospective trial registration was not applicable.

### 2.4 Tools

In this study, a demographic questionnaire, the Cognitive Assessment Scale for Stroke Patients, and the Mini‐Mental State Examination were utilized.

Demographic data and stroke history: This questionnaire was completed based on participant interviews and medical records and included age, sex, educational level, type of stroke, presence or absence of aphasia, lesion location based on magnetic resonance imaging (MRI), and time elapsed since stroke onset.

The Cognitive Assessment Scale for Stroke Patients (CASP): The CASP scale was developed in French by Charles Benaim and colleagues to screen cognitive function in people with stroke, including those with verbal or language impairments. The instrument uses visual and non‐verbal tasks to reduce the influence of language impairment on cognitive assessment. The CASP consists of nine items covering several cognitive functions, including praxis, short‐term memory, temporal orientation, executive function, visuospatial neglect, and language, with a maximum total score of 36 points [5, 8].

The Mini‐Mental State Examination (MMSE): This questionnaire was developed in 1975 by Marshal Folstein for the screening of cognitive decline. The MMSE questionnaire consists of 20 questions, and the total score obtainable from it is 30 points. According to reference texts, a score less than 25 indicates a probable cognitive impairment [12, 13]. This test was translated into Persian in a group of individuals with dementia, who reported its internal reliability based on Cronbach's alpha coefficient as 0.81, and its sensitivity and specificity at a cutoff score of 22 as 90% and 93.5%, respectively [14, 15, 16].

Participants were instructed to complete both questionnaires to the best of their ability; even individuals with aphasia participated in the non‐verbal components of the questionnaires.

In accordance with the original scoring guidelines of the CASP, items that could not be completed due to cognitive or language limitations were scored according to standard administration rules to avoid missing data and ensure consistency.

### 2.5 Translation Process

The CASP was translated into Persian following the standardized forward‐backward translation procedure proposed by Beaton and Dewey [17], which comprises five structured steps. To ensure conceptual and linguistic equivalence, the final translated version was reviewed and approved by the original developer of the instrument. The developer's feedback was carefully incorporated into the Persian version to preserve the integrity of the original scale. Subsequently, the cognitive interview involving 30 participants was conducted to assess the clarity, cultural appropriateness, and comprehensibility of the items. Based on the findings, subtle adjustments were incorporated prior to the finalization of the P‐CASP version. These modifications specifically involved adapting the imagery within the Praxis item to depict a judge and a clergyman in a manner culturally resonant with Iranian sensibilities. Furthermore, the Gregorian calendar item was converted to the Solar (Shamsi) one. Formal authorization to translate and utilize the CASP was obtained from the original author via email.

### 2.6 Second Phase: Psychometric Evaluation

Content validity was evaluated by a panel of 20 experts (occupational therapists, speech‐language pathologists, and neurologists) using the Content Validity Ratio (CVR) based on Lawshe's method and the Item‐ and Scale‐Level Content Validity Index (I‐CVI and S‐CVI).

Discriminant (known‐groups) validity was examined by comparing P‐CASP scores between 60 participants with stroke and 60 age‐ and sex‐matched healthy controls. An independent‐samples *t*‐test was used to assess between‐group differences, and Cohen's *d* was calculated to estimate the magnitude of the group difference. Concurrent validity was assessed by examining the correlation between the CASP and MMSE scores using Pearson's correlation coefficient.

Internal consistency was assessed for the total P‐CASP score using Guttman's lambda‐2 (*λ*2). This coefficient was selected as an alternative estimate of internal consistency given the multidimensional content of the instrument and the limitations in interpreting Cronbach's alpha for such a scale. The total‐score approach was consistent with the one‐dimensional structure and scoring approach reported in the original validation study of the CASP.

Test‐retest reliability was assessed using the Intraclass Correlation Coefficient (ICC), with a 2‐week interval between the two assessments. Only the P‐CASP was re‐administered at the retest session; the MMSE was administered once at baseline for concurrent validity assessment. This period was chosen as it prevents memory bias stemming from the recall of initial responses, while also not being so long as to induce significant changes or advancements in participants' cognitive state.

### 2.7 Responsiveness (Sensitivity to Change) and Minimal Detectable Change (MDC)

To determine the smallest change in P‐CASP scores that exceeds measurement error, the minimal detectable change at the 95% confidence level (MDC_95_) was calculated using a distribution‐based approach. Given the cross‐sectional design of the study and the absence of a global rating of change or a reference standard measured at both time points, anchor‐based MCID estimation was not feasible. Therefore, MDC_95_ is interpreted as a statistical threshold for true change rather than a clinically meaningful improvement.

First, the Standard Error of Measurement (SEM) was estimated using the following formula [18]:

SEM=SD×√(1−ICC)

where SD represents the standard deviation of the baseline P‐CASP scores, and ICC corresponds to the test–retest reliability coefficient.

Subsequently, MDC_95_ was calculated as [19]:

MDC95=1.96×√2×SEM

The MDC95 was interpreted as the minimum change required to exceed measurement error with 95% confidence under the conditions of the present study. Because the study did not include an external anchor or an independent indicator of clinically meaningful change, MDC95 was not interpreted as a Minimal Clinically Important Difference (MCID).

### 2.8 Discriminative Analysis

Receiver operating characteristic (ROC) curve analysis was performed to examine the ability of the P‐CASP scores to discriminate between the stroke and healthy control groups included in the study. The area under the curve (AUC), sensitivity, specificity, and corresponding cutoff value were calculated.

Because the reference classification was based on group membership rather than an independent clinical diagnosis of post‐stroke cognitive impairment, the ROC findings were interpreted as evidence of discrimination between the two study groups and not as evidence of diagnostic accuracy for post‐stroke cognitive impairment.

### 2.9 Statistical Analysis

Statistical analyses were performed using IBM SPSS Statistics version 24.0 (IBM Corp., Armonk, NY, USA). Continuous variables were summarized using the mean and standard deviation (SD), and categorical variables were summarized using frequencies and percentages.

- All statistical tests were two‐sided, and an a priori significance level of *p *< 0.05 was used.
- An independent‐samples *t*‐test was used to compare P‐CASP scores between the stroke and healthy control groups. Welch's correction was applied when the assumption of equal variances was not met.
- Pearson's correlation coefficient was used to assess the association between P‐CASP and MMSE total scores, as both were treated as continuous variables and their distributions were approximately normal.
- Internal consistency of the overall P‐CASP score was assessed using Guttman's lambda‐2 (*λ*2). This coefficient was used as an estimate of internal consistency and was not interpreted as evidence of unidimensionality.
- Test–retest reliability was assessed using an ICC based on a two‐way mixed‐effects model with absolute agreement.
- ROC curve analysis was used to calculate the AUC, sensitivity, specificity, and optimal cutoff value.
- SEM and MDC_95_ were calculated to quantify measurement error.
- Subgroup analyses comparing aphasic and non‐aphasic participants were considered exploratory and were performed to examine whether language impairment influenced the association between P‐CASP and MMSE scores.
- Effect sizes were reported where appropriate, including Cohen's *d* for between‐group comparisons and correlation coefficients for associations.
- Statistical reporting was guided by established recommendations for clinical research statistics [20].

## 3 Results

### 3.1 Demographic Characteristics

In addition to the 30 participants involved in the translation phase, 136 PwS were included in the main study. The mean post‐stroke duration was approximately 3 months, and most participants were over 55 years old. Eighty‐seven participants (64%) were male, and 49 (36%) had aphasia according to medical records. A total of 79 participants (58.1%) experienced their first stroke 2–3 months before enrollment. Lesions were located on the right side in 76 participants (55.9%) and affected the dominant side in 87 participants (64%). In the healthy control group, most participants were over 55 years old (40%), and 34 (56.7%) were male (Table 1). Mean scores were 18.23 ± 10.88 on the CASP and 16.49 ± 11.53 on the MMSE.

**Table 1:** Clinical and demographic characteristics of people with stroke (*N* = 136) and healthy controls (*N* = 60).

Groups	Variable	Frequency	Percent
People with stroke (*N* = 136)	Age (years), mean ± SD	54.17 ± 7.03	—
	Sex		
	Male	87	64.0
	Female	49	36.0
	Education		
	Non‐academic	83	61.0
	Academic	53	39.0
	Aphasia		
	Without	87	64.0
	With	49	36.0
	Period after stroke (days)		
	1–30	6	4.4
	31–60	51	37.5
	61–90	79	58.1
	Dominant hand		
	Non‐dominant	49	36.0
	Dominant	87	64.0
	Location of lesion		
	Right	76	55.9
	Left	56	41.2
	Bilateral	4	2.9
Healthy persons (*N* = 60)	Age (years), mean ± SD	51.50 ± 7.13	—
	Sex		
	Male	34	56.7
	Female	26	43.3

### 3.2 Validity

#### 3.2.1 Content Validity

The Content Validity Ratio (CVR) values for the nine CASP items ranged from 0.70 to 1.00, with all values meeting the minimum acceptable criterion according to Lawshe's method [21]. The Item‐Level Content Validity Index (I‐CVI) values ranged from 0.95 to 1.00, indicating high agreement among experts regarding the relevance of the translated items. These findings supported the content validity of the P‐CASP. Detailed CVR and I‐CVI values are presented in Table 2.

**Table 2:** Content validity of the cognitive assessment scale for people with strokes (20 cognitive experts).

Item	CVR	CVI
1	1	1
2	1	1
3	1	1
4	1	1
5	1	1
6	0.7	1
7	1	1
8	0.9	0.95
9	1	1

### 3.3 I‐CVI, Item‐Level Content Validity Index

#### 3.3.1 Criterion Validity

The Pearson correlation between P‐CASP and MMSE was strong (*r* = 0.886, *p *< 0.001), supporting concurrent validity. Subgroup analyses showed correlations of 0.692 in aphasic participants and 0.942 in non‐aphasic participants, all statistically significant (*p *< 0.001).

#### 3.3.2 Discriminant Validity (Known‐Groups Validity)

Mean CASP scores were 24.43 (SD = 1.64) for participants and 35.23 (SD = 11.03) for controls, a difference of 10.81. Levene's test indicated unequal variances; therefore, Welch's *t*‐test was applied, revealing a statistically significant difference between groups (*t* = 7.508,*p* < 0.001).

The effect size was large (Cohen's *d *= 1.36), indicating a strong ability of the CASP to discriminate between stroke patients and healthy controls (Table 3).

**Table 3:** Discriminant validity of the CASP compared to people with stroke and control groups (*n* = 60).

Groups	*n*	Mean	Standard deviation	Mean difference	Leven' *F*	Leven' *p*	*t*	*p* value	Cohen's
Healthy persons	60	35.23	1.64	10.81	102.145	> 0.001	7.508	> 0.001	1.36
People with Stroke	60	24.43	11.03						

The stroke and healthy control groups differed significantly in CASP scores (Table 3). These findings support the known‐group discrimination of the P‐CASP by demonstrating statistically significant differences between groups expected to differ in cognitive performance.

### 3.4 Reliability

Internal consistency: The Guttman's lambda‐2 (*λ*2) coefficient for the overall 9‐item P‐CASP was 0.922 (*n* = 136), indicating high internal consistency. The *λ*2 coefficient was also calculated separately for participants with aphasia (*λ*2 = 0.892, *n* = 49) and those without aphasia (*λ*2 = 0.881, *n* = 87). These findings indicate high internal consistency across the total sample and both language‐status subgroups.

Test–retest reliability was excellent over the 2‐week interval (ICC(2,1(= 0.997, 95% CI: 0.996–0.998).

### 3.5 Measurement Error and Minimal Detectable Change

The Standard Error of Measurement (SEM) was estimated as 0.60 points based on the test–retest reliability coefficient.

The Minimal Detectable Change at the 95% confidence level (MDC_95_) was calculated using a distribution‐based approach:

$$\mathrm{SEM}=\mathrm{SD}\times \sqrt{1-\mathrm{ICC}}=10.88\times \sqrt{1-0.997}\approx 0.60{\mathrm{MDC}}_{95}=1.96\times \sqrt{2}\times \mathrm{SEM}\approx 1.66$$

This MDC_95_ value of 1.66 points indicates that any change in P‐CASP scores greater than 1.66 can be interpreted as a true change beyond measurement error, while smaller changes are likely due to variability in measurement. Given the cross‐sectional design of the study and the absence of an intervention or longitudinal follow‐up, MDC_95_ should be interpreted as a statistical threshold for true change, rather than a clinically observed improvement.

### 3.6 Feasibility in Participants With Aphasia

Feasibility of administration was examined among the 49 participants with aphasia. Feasibility was operationally defined as the ability to obtain a scorable result. Using the predefined cutoff of 5 points, 8 participants (16.3%) scored below this threshold on the P‐CASP, compared with 41 participants (83.7%) on the MMSE. These findings suggest that a greater proportion of participants with aphasia were able to obtain scorable P‐CASP results than MMSE results.

### 3.7 Discriminative Analysis

**Figure 1:**
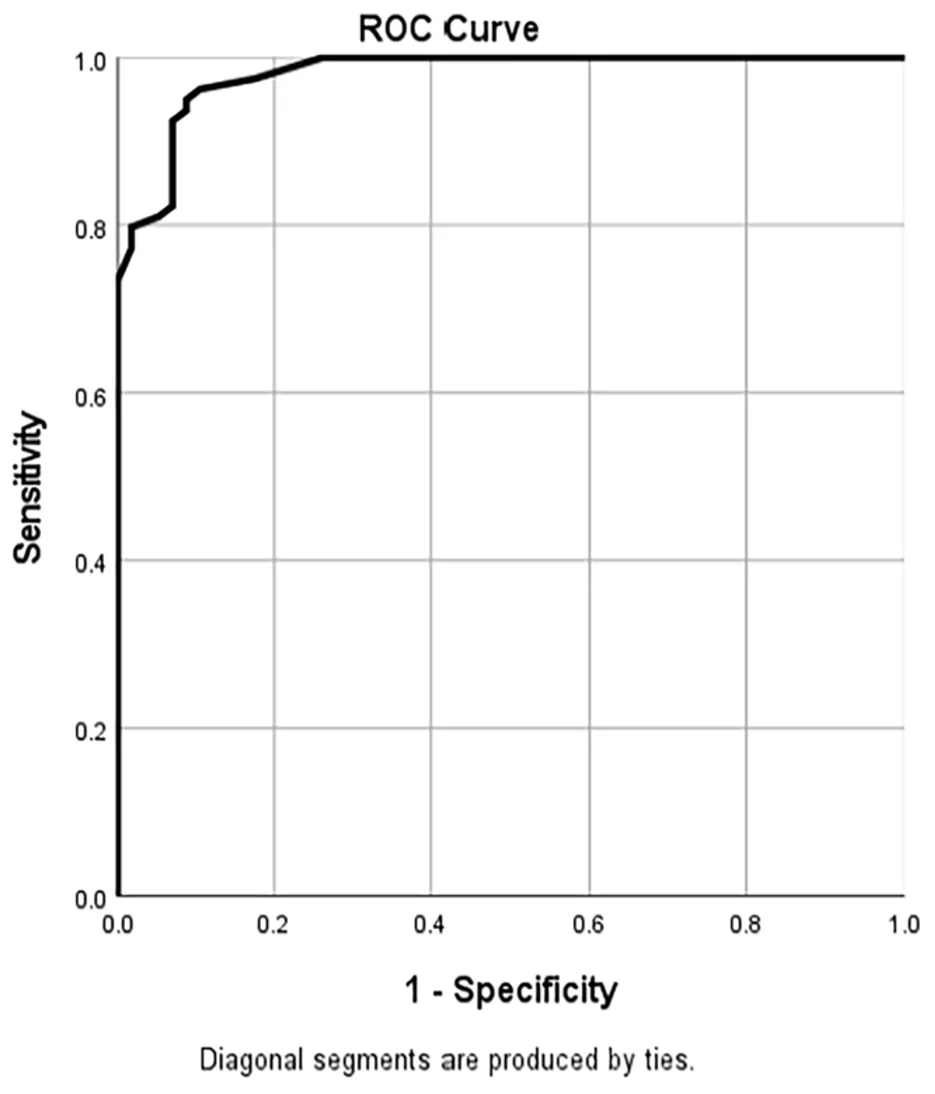
ROC curve of the CASP cognitive tool sensitivity. (AUC value is 0.98; CI, 0.93–0.98 95%) Abbreviations: AUC, area under the curve; ROC, receiver operating characteristic.

ROC analysis demonstrated excellent diagnostic performance of the CASP (AUC = 0.980, SE = 0.009, *p *< 0.001) in distinguishing individuals with cognitive impairment from healthy controls. Based on the ROC coordinates and prioritizing sensitivity for screening purposes, a cutoff of 31.25 was selected. At this threshold, sensitivity was 100%, and specificity was 73.7%. Lower CASP scores indicate a higher probability of cognitive impairment, making this threshold suitable for identifying potential cases (Figure 1).

## 4 Discussion

This study translated and culturally adapted the Cognitive Assessment Scale for Stroke Patients (CASP) into Persian (P‐CASP) and evaluated its psychometric properties in people with stroke (PwS). Overall, the P‐CASP demonstrated strong validity, high internal consistency, excellent test–retest reliability, and good discrimination between stroke and healthy control groups. These findings are broadly consistent with previous evaluations of the French, Italian, and Korean versions of the CASP [9, 10, 22] and support its applicability in Persian‐speaking populations.

Administration feasibility was a notable advantage of the P‐CASP, particularly among individuals with language impairments. Consistent with Barnay et al. [5], fewer administration difficulties were observed with the CASP than with the MMSE in participants with aphasia. In our sample, only 16.3% of aphasic participants scored below 5 on the P‐CASP, compared with 83.7% on the MMSE. This finding highlights the practical value of the P‐CASP in individuals with language impairment and may be related to its predominantly visual and non‐verbal format, which reduces dependence on verbal responses.

Content validity findings indicated that the P‐CASP items were relevant and representative of the cognitive domains assessed by the original CASP, with high agreement among the expert panel [10]. The instrument covers multiple stroke‐relevant domains, including memory, praxis, visuospatial abilities, temporal orientation, executive function, and neglect. These features support the appropriateness of the translated items for assessing cognitive function in PwS.

Concurrent validity was demonstrated through strong correlations between P‐CASP and MMSE scores (*r* = 0.886, *p *< 0.001). The correlation was somewhat lower in aphasic participants (*r* = 0.692), likely due to language impairments affecting test performance, whereas non‐aphasic participants showed a very strong correlation (*r* = 0.942).

The lower correlation observed in participants with aphasia may reflect differences in language demands between the two instruments. This finding supports the concurrent validity of P‐CASP while suggesting that it may be less influenced by language impairment than the MMSE.

Furthermore, the inclusion of items assessing unilateral spatial neglect, praxis, and executive function, which are not addressed by MMSE, enhances P‐CASP's sensitivity in detecting cognitive deficits specific to stroke. These features may increase its relevance for detecting stroke‐related cognitive deficits, particularly in individuals with aphasia.

Known‐group validity was supported by the finding that stroke participants scored significantly lower than matched healthy controls (*t* = 7.508, *p *< 0.001; Cohen's *d* = 1.36), indicating a large between‐group difference and supporting the ability of the P‐CASP to discriminate between the two groups. Internal consistency of the total P‐CASP was high, as indicated by a Guttman's lambda‐2 coefficient of 0.922. The *λ*2 coefficients were also high among participants with aphasia (0.892) and those without aphasia (0.881), indicating consistent item performance across the total sample and language‐status subgroups. Test–retest reliability over a 2‐week interval was excellent (ICC = 0.997), demonstrating high temporal stability.

The near‐perfect test–retest reliability observed in the present study (ICC = 0.997) may be attributed to several methodological and clinical factors. Primarily, the 2‐week interval between assessments was selected to balance the potential for recall bias with the possibility of true cognitive changes or spontaneous neurological recovery during the subacute phase of stroke. Furthermore, the CASP uses a highly structured administration protocol with explicit scoring criteria and a predominantly visual format, which may reduce administration‐related variability across repeated measurements and contribute to the observed temporal stability of the scores.

The use of Guttman's lambda‐2 was intended to provide an estimate of internal consistency without interpreting the coefficient as evidence of unidimensionality. The dimensional structure of the CASP was previously investigated in the original validation study [10], which supported a one‐dimensional structure and the use of an overall score. Nevertheless, further studies using factor‐analytic approaches in Persian‐speaking samples would be valuable to independently examine the dimensional structure of the P‐CASP.

The Minimal Detectable Change at the 95% confidence level (MDC_95 _= 1.66) represents the minimum change required to exceed measurement error under the conditions of the present study. Receiver Operating Characteristic (ROC) analysis demonstrated excellent discrimination between the stroke and healthy (AUC = 0.980). Using a cutoff score of 31.25, the P‐CASP achieved a sensitivity of 100% and a specificity of 73.7% for distinguishing the stroke and healthy control groups included in this study [10].

Overall, these findings support the validity, reliability, and feasibility of the P‐CASP as a cognitive screening instrument for Persian‐speaking people with stroke. Its visual and language‐independent design may facilitate assessment in individuals with aphasia. Further longitudinal studies are needed to evaluate responsiveness to clinical change and broader clinical applicability.

### 4.1 Limitations

This study has several limitations. First, convenience sampling was used because a comprehensive stroke registry was unavailable, which may limit generalizability and introduce selection bias. Second, only individuals aged 40–60 years with a first‐ever unilateral ischemic stroke were included to reduce clinical heterogeneity and age‐related confounding; therefore, findings may not generalize to patients with hemorrhagic or recurrent stroke or to older adults. Third, participants with aphasia could not be classified by aphasia subtype because of limited access to detailed clinical information. Furthermore, because no validated depression measure specifically designed for individuals with aphasia was available, the BDI was administered to all participants, which may have limited the accuracy of depression screening in this subgroup.

Fourth, the MMSE was administered only at baseline and was not repeated during test–retest assessment; therefore, an external anchor or anchor‐based Minimal Clinically Important Difference (MCID) could not be established. Future longitudinal studies should incorporate appropriate external anchors to determine clinically meaningful change thresholds for the P‐CASP.

Finally, although the CASP assesses multiple cognitive domains, internal consistency was evaluated at the total‐score level, consistent with the scoring structure and one‐factor solution reported in the original validation study. However, the dimensional structure of the Persian version was not independently examined using factor analysis. Thus, the internal consistency coefficient should not be interpreted as evidence of unidimensionality. Future studies should confirm the dimensional structure of the P‐CASP using exploratory or confirmatory factor analysis and evaluate additional model‐based reliability estimates where appropriate.

### 4.2 Recommendations for Future Research

Future studies should further examine the dimensional structure of the P‐CASP using exploratory or confirmatory factor analysis and evaluate additional model‐based reliability estimates where appropriate. They should also assess the performance of the P‐CASP in stroke patients with different types and severity levels of aphasia to determine its reliability and accuracy across diverse clinical profiles. In addition, examining the correlation between the P‐CASP and the Montreal Cognitive Assessment (MoCA) is recommended to further establish its convergent validity.

## 5 Conclusion

The results of this study indicate that the Persian version of CASP can be used as a highly reliable, efficient, and suitable tool for screening cognitive impairments in PwS with acute stroke. Given that the purpose of this test is screening, it is recommended that PwS who score within the pathological range on this test be referred to a neurologist for further specialized evaluation and treatment.

## Author Contributions

**Farzad Hekmatifar:** conceptualization, investigation, writing – original draft, formal analysis, visualization. **Nazila Akbarfahimi:** project administration, supervision, writing – original draft, methodology. **Mohammad Sayadnasiri:** validation, methodology, supervision. **Razieh Bidhendi‐Yarandi:** methodology, formal analysis, data curation, software. **Samira Rostami:** investigation. **Negin Fallahi‐Khoshknab:** writing – review and editing. **Charles Benaim:** validation, writing – review and editing.

## Funding

The authors have nothing to report.

## Conflicts of Interest

The authors declare no conflicts of interest.

## Transparency Statement

The corresponding author, Nazila Akbarfahimi, affirms that this manuscript is an honest, accurate, and transparent account of the study being reported, that no important aspects of the study have been omitted; and that any discrepancies from the study as planned (and, if relevant, registered) have been explained.

## Data Availability

The data that support the findings of this study are available from the corresponding author upon reasonable request. Data are not publicly available due to privacy and ethical restrictions on participant information.
